# Lumbar spine fusion outcomes using a cellular bone allograft with lineage-committed bone-forming cells in 96 patients

**DOI:** 10.1186/s12891-021-04584-z

**Published:** 2021-08-17

**Authors:** Hossein Elgafy, Bradley Wetzell, Marshall Gillette, Hassan Semaan, Andrea Rowland, Christopher A. Balboa, Thomas A. Mierzwa, Julie B. McLean, Kimberly Dorsch, Mark A. Moore

**Affiliations:** Department of Orthopaedic Surgery, University of Toledo Medical Center, 3065 Arlington Avenue, Toledo, OH 43614 USA

**Keywords:** Cellular bone allograft, CBA, Lumbar fusion, Instrumented posterior lumbar fusion, IPLF, TLIF, Transforaminal interbody fusion, ViviGen

## Abstract

**Background:**

Instrumented posterior lumbar fusion (IPLF) with and without transforaminal interbody fusion (TLIF) is a common treatment for low back pain when conservative interventions have failed. Certain patient comorbidities and lifestyle risk factors, such as obesity and smoking, are known to negatively affect these procedures. An advanced cellular bone allograft (CBA) with viable osteogenic cells (V-CBA) has demonstrated high fusion rates, but the rates for patients with severe and/or multiple comorbidities remain understudied. The purpose of this study was to assess fusion outcomes in patients undergoing IPLF/TLIF using V-CBA with baseline comorbidities and lifestyle risk factors known to negatively affect bone fusion.

**Methods:**

This was a retrospective study of de-identified data from consecutive patients at an academic medical center who underwent IPLF procedures with or without TLIF, and with V-CBA. Baseline patient and procedure characteristics were assessed. Radiological outcomes included fusion rates per the Lenke scale. Patient-reported clinical outcomes were evaluated via the Oswestry Disability Index (ODI) and Visual Analog Scale (VAS) for back and leg pain. Operating room (OR) times and intraoperative blood loss rates were also assessed.

**Results:**

Data from 96 patients were assessed with a total of 222 levels treated overall (mean: 2.3 levels) and a median follow-up time of 16 months (range: 6 to 45 months). Successful fusion (Lenke A or B) was reported for 88 of 96 patients (91.7%) overall, including in all IPLF-only patients. Of 22 patients with diabetes in the IPLF+TLIF group, fusion was reported in 20 patients (90.9%). In IPLF+TLIF patients currently using tobacco (*n* = 19), fusion was reported in 16 patients (84.3%), while in those with a history of tobacco use (*n* = 53), fusion was observed in 48 patients (90.6%). Successful fusion was reported in all 6 patients overall with previous pseudarthrosis at the same level. Mean postoperative ODI and VAS scores were significantly reduced versus preoperative ratings.

**Conclusion:**

The results of this study suggest that V-CBA consistently yields successful fusion and significant decreases in patient-reported ODI and VAS, despite patient comorbidities and lifestyle risk factors that are known to negatively affect such bony healing.

## Background

Low back pain is among the most prevalent medical complaints across the globe and a leading cause of disability [1]. While some patients find relief from noninvasive interventions, spine surgery may be indicated when these treatments are not successful. Instrumented posterior lumbar fusion (IPLF) is one such surgical procedure often performed for spondylolisthesis, degenerative lumbar disc, and facet arthropathy [2]. In recent years, some studies have found improved fusion and functional outcomes with the addition of interbody devices, such as with transforaminal interbody fusion (TLIF), leading to their increased use [3, 4]. Although generally successful, IPLF/TLIF surgeries are known to be negatively affected by certain patient comorbidities and lifestyle risk factors, such as obesity and smoking, which can slow or prevent fusion [5–7].

Successful bone fusion requires three main properties: an osteoconductive scaffold to support it, osteoinductive molecular signals to promote it, and osteogenic cells to facilitate it [8]. Autologous bone, such as iliac crest bone graft (ICBG), is the traditionally-preferred source of these properties [8]. However, its quality is inherently limited by patient age, comorbidities, and lifestyle risk factors [9]. Further, the additional surgical procedure to harvest ICBG increases operative time and blood loss, with subsequent increases in cost and postoperative pain. Local bone from the primary surgical site is another common graft option, but its available volume is limited, and it remains, on its own, subject to the same patient-related limitations as ICBG [10]. As a result, numerous alternatives to autograft bone, including allogeneic bone, have emerged with the goal of facilitating bone formation while limiting the inherent drawbacks of autograft.

Among these allogeneic alternatives, cellular bone allografts (CBAs) are a relatively new class of bone void filler that are designed to preserve viable osteogenic cells within an osteoconductive corticocancellous bone matrix and also contain demineralized bone to enhance osteoinductivity [11–13]. Thus, CBAs can theoretically provide all three necessary properties of bone formation, potentially providing the benefits of autologous bone grafts while minimizing their inherent drawbacks. However, the majority of available CBAs purport to rely on mesenchymal stem cells (MSCs) to develop into an osteogenic component. While MSCs have the potential to differentiate into osteogenic cells, the process is time consuming and dependent upon local molecular signals which, similar to autograft, may be impaired by patient age, comorbidities, and lifestyle risk factors [9]. In these cases, MSCs may also differentiate into unwanted cell types, such as adipocytes or myocytes, which could inhibit or delay bone formation and complicate fusion.

A more advanced CBA has thus been developed to reduce this uncertainty by preserving viable native lineage-committed osteogenic cells (ViviGen®; V-CBA; LifeNet Health®, Virginia Beach VA), which have been shown both preclinically and clinically to outperform MSCs in bone formation [14–18]. While high fusion rates have been previously reported in IPLF procedures using V-CBA [10], the fusion rate for patients with severe and/or multiple comorbidities remains understudied. Therefore, the purpose of this study was to retrospectively assess clinical outcomes in patients undergoing IPLF surgeries with and without TLIF and using V-CBA, who had baseline comorbidities and lifestyle risk factors known to negatively affect bone fusion.

## Methods

### Patients and variables

This was a retrospective study of de-identified data from consecutive patients undergoing IPLF procedures with or without TLIF performed by the first author (HE) using V-CBA at an academic medical center from January 2016 to November 2018. Criteria for inclusion were patients at least 18 years of age at the time of surgery and with indication of lumbar spine fusion for degenerative changes including spondylolisthesis, disc degeneration and facet arthropathy, or revision for pseudarthrosis. Criteria for exclusion were patients with infection, trauma, and/or tumor. The protocol for this study was approved by the first author’s institutional review board (University of Toledo Protocol Number: 202855-UT).

Baseline patient and procedure characteristics that were assessed included age; sex; race/ethnicity; body mass index (BMI; overall and incidences of patients either below or at least 30 kg/m^2^); incidences of diabetes, tobacco use (current and history), and cancer; distributions of presurgical pain and treatments prescribed prior to surgery; and number of levels treated (overall and distribution of each). Continuous variables were summarized as means and standard deviations (SDs) and categorical variables were summarized as numbers and percentages of all patients.

Radiological outcomes that were assessed included fusion rates (see Assessment of fusion, below) by treatment and number of levels treated, last visit, and baseline risk factors, which were summarized as the percentage of patients within each treatment and respective category. Clinical outcomes included patient-reported pre- versus postoperative percentage of disability per the Oswestry Disability Index (ODI) [19], and pre- versus postoperative back and leg pain using the Visual Analog Scale (VAS) [20], which were summarized as means and standard error of the means (SEMs) and compared using two-sided paired *T*-tests. Postoperative ODI and VAS data were collected at the last visit. Operating room (OR) time (in minutes) and intraoperative blood loss (in mL) were also summarized as means and SDs overall and by number of levels treated.

Statistical assessments were conducted using Prism Version 8.3.0 (GraphPad Software; San Diego CA; www.graphpad.com) and significance was evaluated at the 0.05 alpha level.

### Surgical procedure

All study patients underwent IPLF procedures with or without TLIF, and with V-CBA. The instrumentation used was the Universal Spine System™ (DePuy Synthes, Raynham MA) and a structural interbody allograft spacer (VertiGraft®; LifeNet Health®, Virginia Beach VA) was used for the TLIF procedures. Local bone autograft harvested from the decompression was mixed with V-CBA at varying ratios and placed on both sides of the spine.

### Assessment of fusion

As described previously [21], standing anteroposterior and lateral radiographs and computerized tomography (CT) scans conducted at the last follow-up visit were reviewed by an independent senior musculoskeletal radiologist (HS). The following grading scale was utilized to assess posterolateral fusion as formerly described by Lenke et al. [22]: Grade A, “definitely solid [with] big trabeculated bilateral fusion masses”; Grade B, “possibly solid [with] unilateral large fusion mass [and] a contralateral small fusion mass”; Grade C, “probably not solid [with] small, thin fusion masses bilaterally [and probable unilateral pseudarthrosis]”; and Grade D, “definitely not solid [with] graft resorption bilaterally or fusion mass with obvious bilateral pseudarthrosis”. Grades of A and B were considered fused, and Grades of C and D were considered not fused. Overall fusion ratings reflected the lowest rating at any individual level.

## Results

Baseline patient and procedure characteristics are presented in Table 1. A total of 96 patients were assessed (IPLF *n* = 13; IPLF+TLIF *n* = 83) with a mean age (SD) of 58.9 (11.4) years (IPLF = 64.9 [10.2] years; IPLF+TLIF = 57.9 [11.3] years). Overall, the majority of patients were female (51 patients; 53.1%), Caucasian (77 patients; 80.2%), and classified as obese (ie, BMI ≥30 kg/m^2^; 69 patients; 71.9%), with an overall mean (SD) BMI of 33.7 (6.6) kg/m^2^. A total of 24 patients (25.0%) had diabetes, 21 patients (21.9%) were current tobacco users, 61 patients (63.5%) had a history of tobacco use, and 7 patients (7.3%) had a history of cancer. Among IPLF-only patients, the majority were male (8 patients; 61.5%), Caucasian (10 patients; 76.9%), and classified as obese (10 patients; 76.9%), with a mean (SD) BMI of 35.3 (6.6) kg/m^2^. In this group, 2 patients (15.4%) had diabetes, 2 patients (15.4%) were current tobacco users, 8 patients (61.5%) had a history of tobacco use, and 1 patient (7.7%) had a history of cancer. Among IPLF+TLIF patients, the majority were female (46 patients; 55.4%), Caucasian (67 patients; 80.7%), and classified as obese (59 patients; 71.1%), with a mean (SD) BMI of 33.4 (6.6) kg/m^2^. In the IPLF+TLIF group, 22 patients (26.5%) had diabetes, 19 patients (22.9%) were current tobacco users, 53 patients (63.9%) had a history of tobacco use, and 6 patients (7.2%) had a history of cancer. All patients reported low back and radicular pain, for which conservative management prior to surgery had failed. An overall total of 36 patients (37.5%) had previously undergone lumbar spine surgery: Index revision surgery for pseudarthrosis was performed in 6 patients (6.3%) overall, adjacent or other segment degeneration in 9 patients (9.4%), and 21 patients (21.9%) had non-fusion lumbar surgical procedures, such as microdiscectomy and laminectomy for decompression. An overall total of 222 levels were treated (mean 2.3 levels per patient), with the majority of procedures involving 2 levels (48 patients; 50.0%) or 3 levels (26 patients; 27.0%).

**Table 1:** Baseline Patient and Procedure Characteristics

Characteristic, unit	IPLF Only (***n*** = 13)	IPLF + TLIF (***n*** = 83)	Overall (***N*** = 96)
Age in years, mean (SD)	64.9 (10.2)	57.9 (11.3)	58.9 (11.4)
Sex, n (%)			
-Male	8 (61.5)	37 (44.6)	45 (46.9)
-Female	5 (38.46)	46 (55.4)	51 (53.1)
Race/ethnicity, n (%)			
-Caucasian	10 (76.9)	67 (80.7)	77 (80.2)
-Black or African American	3 (23.1)	12 (14.5)	15 (15.6)
-Hispanic	0 (0.00)	4 (4.8)	4 (4.2)
Body mass index in kg/m^2^, mean (SD)	35.3 (6.6)	33.4 (6.6)	33.7 (6.6)
< 30 kg/m^2^, n (%)	3 (23.1)	24 (28.9)	27 (28.1)
≥ 30 kg/m^2^, n (%)	10 (76.9)	59 (71.1)	69 (71.9)
Diabetes, n (%)	2 (15.4)	22 (26.5)	24 (25.0)
Tobacco use, n (%)			
-Current	2 (15.4)	19 (22.9)	21 (21.9)
-History	8 (61.5)	53 (63.9)	61 (63.5)
Cancer history, n (%)	1 (7.7)	6 (7.2)	7 (7.3)
Distribution of pain, n (%)			
-Back pain with bilateral radiculopathy	2 (15.4)	9 (10.8)	11 (11.5)
-Back pain with right radiculopathy	6 (46.2)	32 (38.6)	38 (39.6)
-Back pain with left radiculopathy	5 (38.4)	40 (48.2)	45 (46.8)
-Back pain	0 (0.00)	2 (2.4)	2 (2.1)
Treatments prior to surgery, n (%)			
-Activity modification	0 (0.0)	1 (1.2)	1 (1.0)
-Brace	0 (0.0)	1 (1.2)	1 (1.0)
-Chiropractor	1 (7.7)	0 (0.0)	1 (1.0)
-None	2 (15.4)	11 (13.3)	13 (13.5)
-Physical therapy	8 (61.5)	60 (72.3)	68 (70.8)
-Prior lumbar surgery (all types)	8 (61.5)	28 (33.7)	36 (37.5)
-Prior fusion surgery, same level(s) (ie, pseudarthrosis)	1 (7.7)	5 (6.0)	6 (6.3)
-Prior fusion surgery, adjacent or other level	1 (7.7)	8 (9.6)	9 (9.4)
-Other prior lumbar surgery	6 (46.2)	15 (18.1)	21 (21.9)
-Spinal injections	1 (7.7)	6 (7.2)	7 (7.3)
-Stretching	2 (15.4)	3 (3.6)	5 (5.2)
-Weight loss	0 (0.0)	1 (1.2)	1 (1.0)
No. levels treated, n (%)			
-1	1 (7.6)	13 (15.7)	14 (14.6)
-2	5 (38.5)	43 (51.8)	48 (50.0)
-3	2 (15.4)	24 (28.9)	26 (27.0)
-4	3 (23.1)	3 (3.6)	6 (6.3)
-5	2 (15.4)	0 (0.0)	2 (2.1)
-All levels, n (mean)	39 (3.0)	183 (2.2)	222 (2.3)

*Abbreviation*: *SD* Standard deviation

Percentages were based on the total number of patients within each treatment or overall

**Fig. 1:**
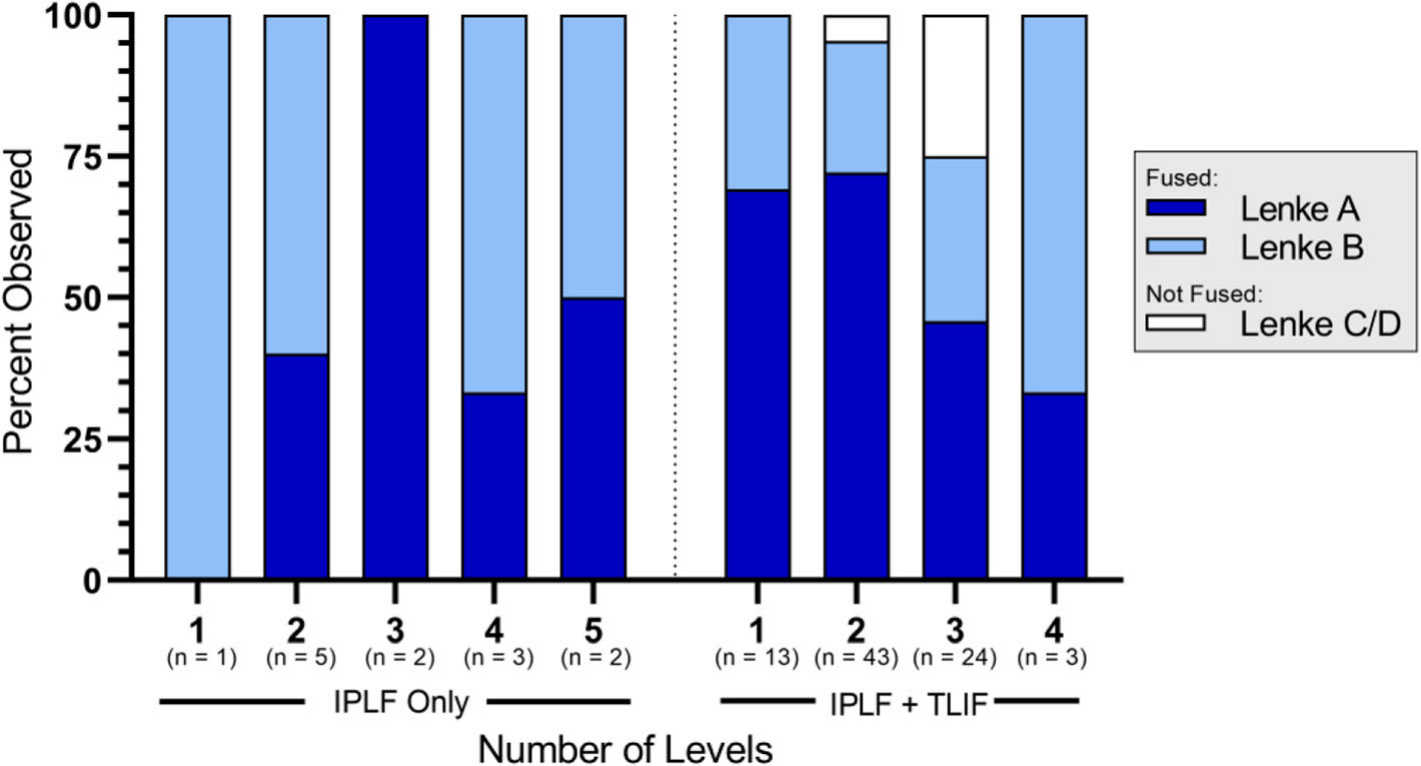
Fusion status by treatment and number of levels treated. Percentages were based on the number of patients within each category

**Fig. 2:**
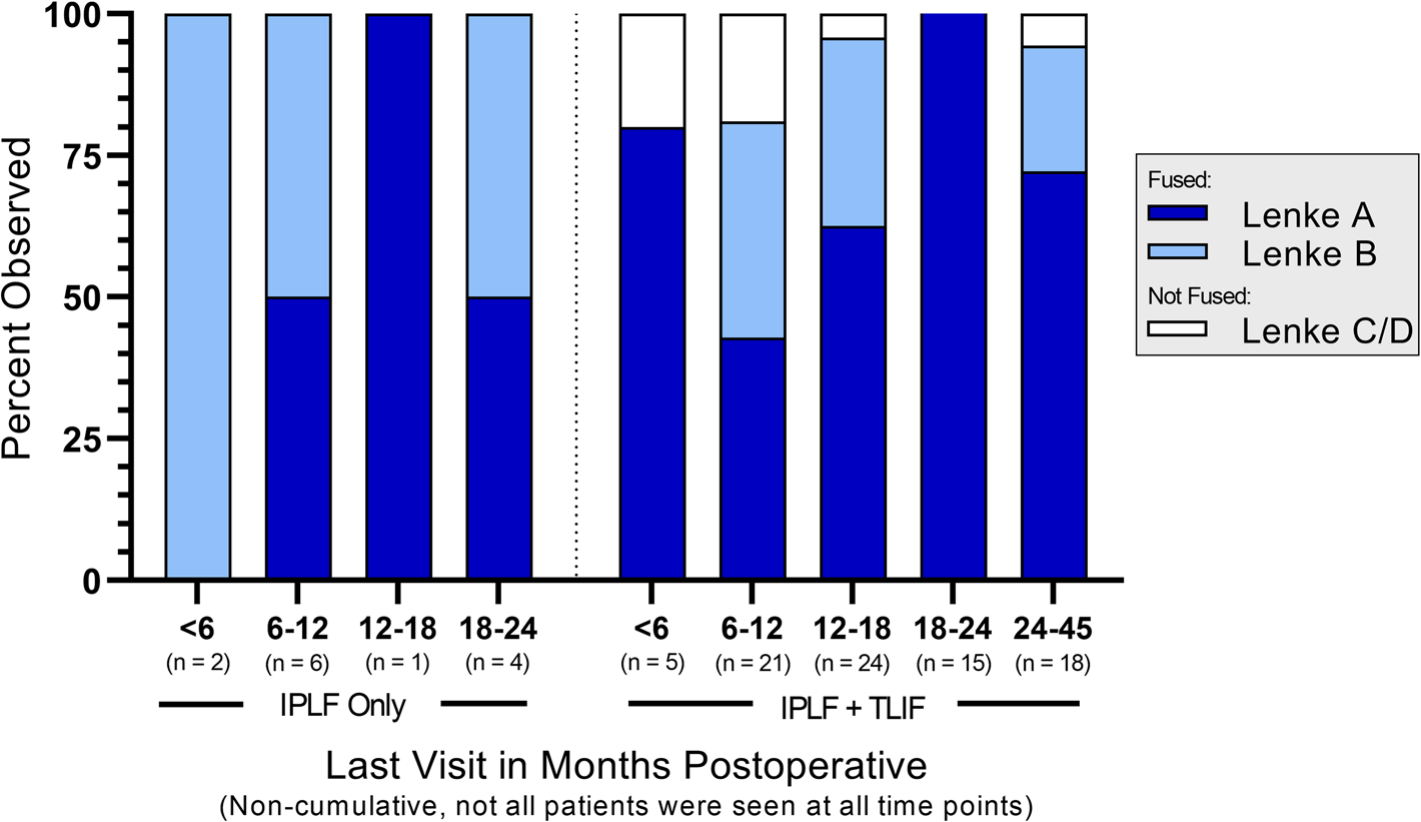
Fusion status by treatment and last visit across all numbers of levels treated. Percentages were based on the number of patients within each category

**Fig. 3:**
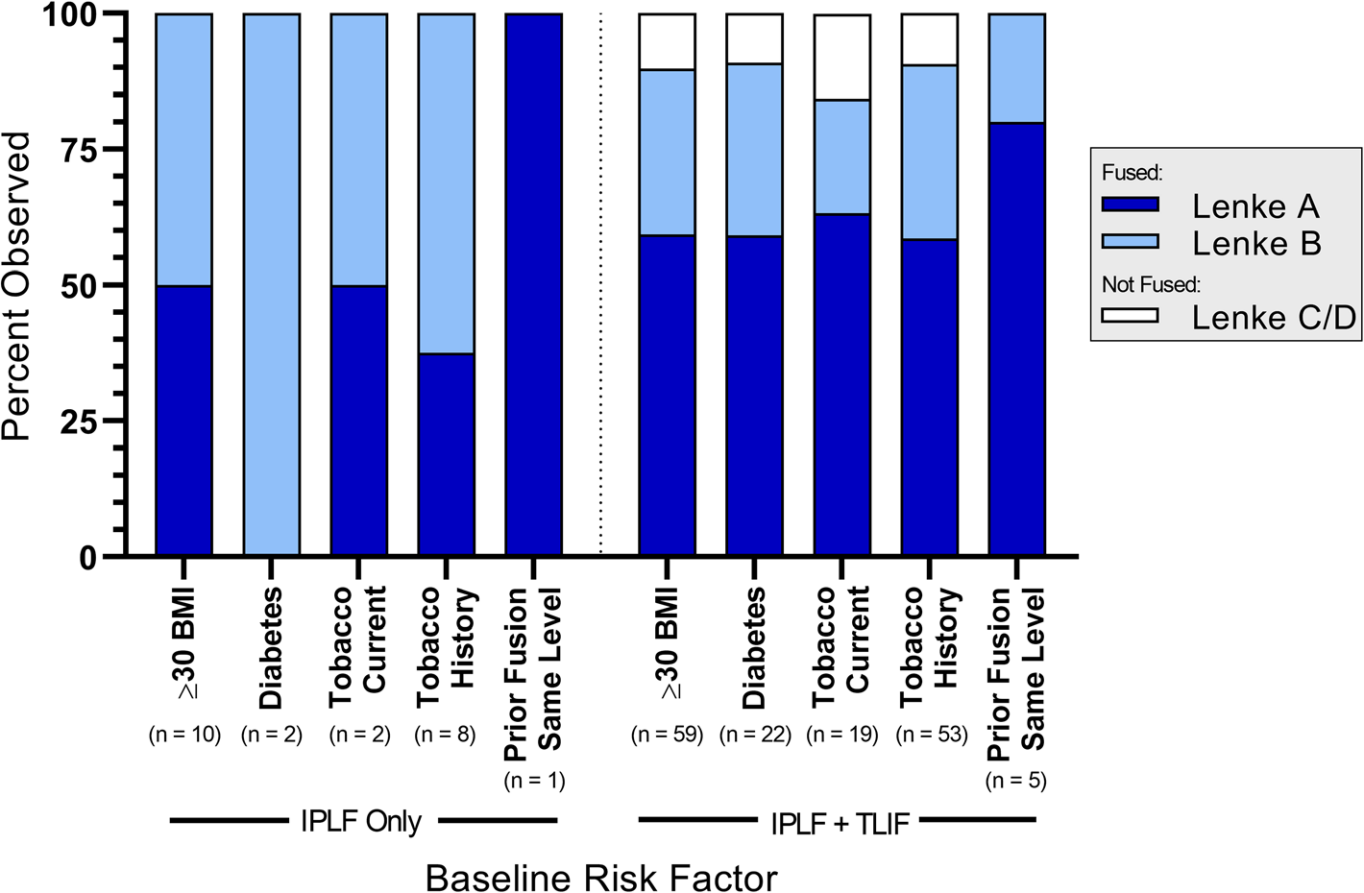
Fusion status by treatment and baseline risk factor as applicable across all numbers of levels treated. Percentages were based on the number of patients within each category

**Fig. 4:**
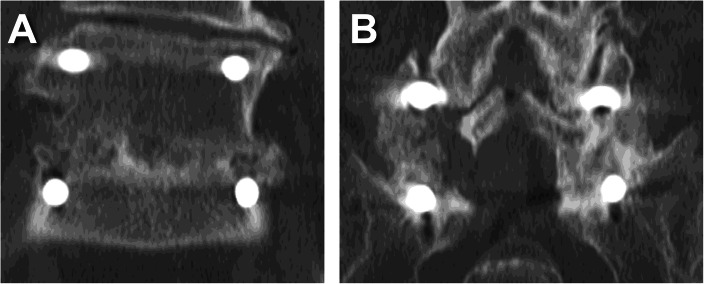
Representative coronal CT scans of a male patient in his 60s at two years postoperative showing **A** bridging callous across the L5-S1 interbody fusion and **B** posterolateral fusion mass (Lenke B, probably solid with a unilateral stout fusion mass and a contralateral thin fusion mass)

**Fig. 5:**
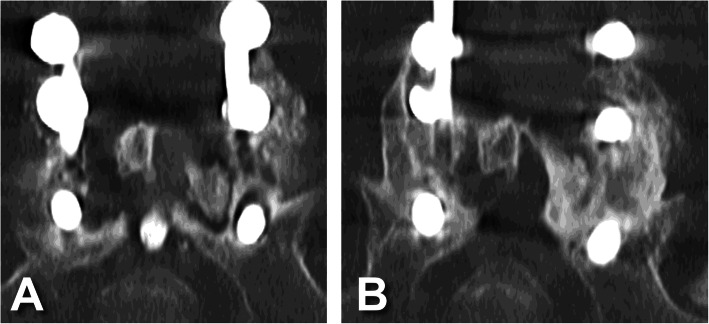
Representative CT scans of a female patient who is a smoker in her 30s. **A** Preoperative coronal view showing loose S1 pedicle screws with halo around the screw track and no posterolateral fusion mass (Lenke D, definitely not solid with thin fusion masses bilaterally with obvious pseudarthrosis). **B** Two years postoperative showing posterolateral fusion mass (Lenke A, bilateral stout fusion masses present)

Fusion status is summarized by treatment and number of levels treated in Fig. 1, by treatment and last visit in Fig. 2, and by treatment and baseline risk factor in Fig. 3. The reported follow-up times ranged from 6 to 45 months with a median of 16 months. Overall, successful fusion (Lenke A or B) was reported for 88 of 96 patients (91.7%; representative CT scans are presented in Figs. 4 and 5), with Lenke C ratings reported for 2 patients (2.1%) and Lenke D ratings reported for 6 patients (6.2%). All patients in the IPLF group were reported as successfully fused (including those with baseline risk factors), with reported Lenke C and D (non-fused) ratings observed only in the IPLF+TLIF group. Among patients with a BMI of at least 30 kg/m^2^ in the IPLF+TLIF group (*n* = 59), successful fusion was reported in 53 patients (89.8%), with Lenke C or D ratings reported for 6 patients (10.2%). Of the 22 patients with comorbid diabetes in the IPLF+TLIF group, Lenke A or B ratings were reported in 20 patients (90.9%) and Lenke C or D ratings were reported in 2 patients (9.1%). In patients currently using tobacco in the IPLF+TLIF group (*n* = 19), successful fusion was reported in 16 patients (84.3%) and non-fusion was reported in 3 patients (15.7%), while in those reporting a history of tobacco use (*n* = 53), fusion was observed in 48 patients (90.6%) and 5 patients (9.4%) did not fuse. Successful fusion was reported in all 6 patients overall receiving treatment for pseudarthrosis at the same level.

Characteristics of the 8 patients considered not fused (Lenke C or D) in the IPLF+TLIF group are summarized in Table 2. These patients ranged in age from 34 to 85 years, and the majority were males (*n* = 6), Caucasian (*n* = 6), and with BMIs of 30 kg/m^2^ or more (n = 6). Two patients in this group were reported to have comorbid diabetes, 3 patients were current tobacco users, 2 patients had a history of tobacco use, and 1 patient had a history of breast cancer. None of these patients had prior fusion surgery or a current diagnosis of pseudarthrosis. Finally, although available patient-reported pre- to postoperative VAS remained relatively consistent, all such patients reported reductions between 3 and 35% in postoperative ODI compared with preoperative ratings.

**Table 2:** Summary of Patients with Lenke C or D Radiological Outcomes

Patient ID	Age (Years)	Sex	Race/Ethnicity	BMI (kg/m^**2**^)	Diabetes	Tobacco	Cancer History	Prior Lumbar Surgery	TLIF	No. Levels	Lenke	ODI		VAS	
												Pre	Post	Pre	Post
001–013	62	F	Black or African American	40.3	N	None	Y (Breast)	N	Y	3	C	47	12	–	6
001–038	44	M	Caucasian	33.63	N	Current	N	Microdiscectomy, Same level(s)	Y	2	D	43	35	8	8
001–039	41	F	Caucasian	45.1	N	Current	N	N	Y	3	D	–	–	–	7
001–054	34	M	Black or African American	42.1	Y	Current	N	N	Y	2	C	45	19	8	6
001–063	62	M	Caucasian	20.0	N	History	N	N	Y	3	D	–	–	–	6
001–090	68	M	Caucasian	25.1	Y	None	N	N	Y	3	D	33	30	6	6
001–137	85	M	Caucasian	36.6	N	None	N	N	Y	3	D	34	9	7	8
001–196	68	M	Caucasian	31.9	N	History	N	N	Y	3	D	43	38	8	7

**Fig. 6:**
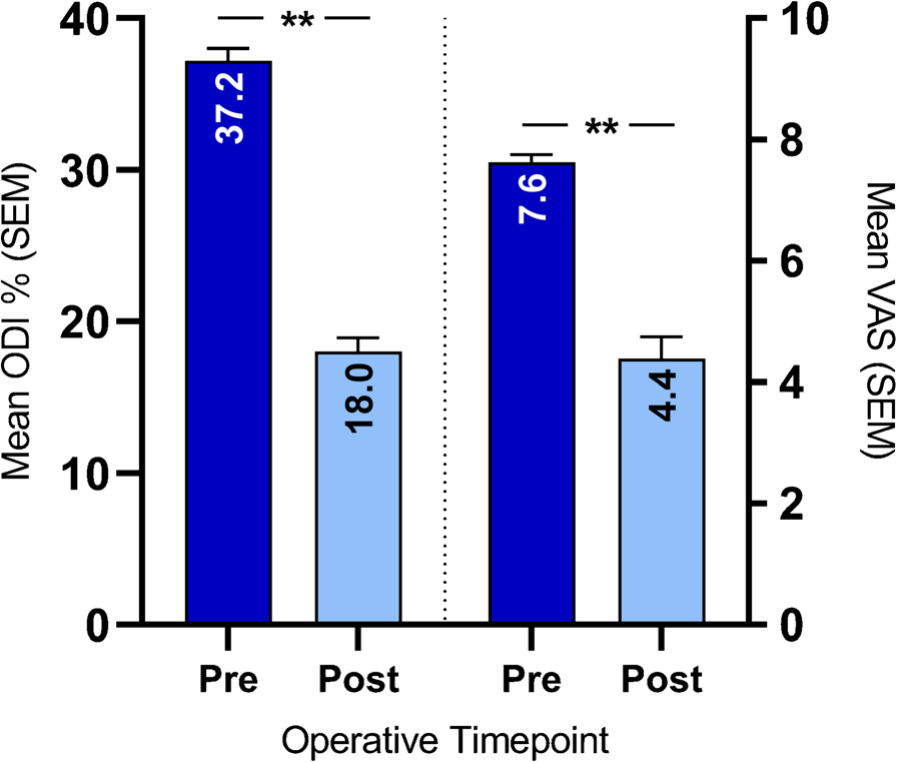
Overall mean (SEM) patient-reported pre- versus postoperative ODI and VAS ratings. ODI data were collected from 76 of 96 patients (79.2%). VAS data were collected from 75 of 96 patients (78.1%). Postoperative data were collected at the last visit. **P < 0.0001, two-sided paired *T*-tests

Overall mean (SEM) pre- versus postoperative ODI and VAS ratings are summarized in Fig. 6. ODI data were reported for both timepoints from 76 of 96 patients (79.2%) and complete VAS data were reported from 75 of 96 patients (78.1%). Mean (SEM) postoperative ODI (18.0 [0.91]) was significantly lower than preoperative ODI (37.2 [0.83]; *P* < 0.0001), as was mean postoperative VAS (4.4 [0.36]) compared with preoperative VAS (7.6 [0.13]; *P* < 0.0001).

Mean (SD) OR time and blood loss are summarized by treatment and number of levels treated in Table 3. Mean OR time ranged from 179.4 to 307.0 min overall, with only slight linear correspondence to number of levels treated. The overall mean (SD) OR time for all levels treated was 192.7 (53.8) minutes. Mean blood loss ranged from 376.2 to 800.0 mL overall, with only slight linear correspondence to number of levels treated. The overall mean (SD) blood loss for all levels treated was 531.9 (350.1) mL.

**Table 3:** Summary of Operating Room Time and Intraoperative Blood Loss

Factor, unit No. levels treated	IPLF Only (***n*** = 13)	IPLF + TLIF (***n*** = 83)^a^	Overall (***N*** = 96)
Operating room time, mean minutes (SD)			
-1	235.0 (−)	175.2 (33.1)	179.4 (35.6)
-2	181.0 (42.9)	168.5 (43.0)	169.8 (42.7)
-3	195.5 (0.7)	231.7 (54.4)	228.8 (53.1)
-4	204.3 (21.4)	220.0 (31.2)	212.2 (25.4)
-5	307.0 (94.8)	–	307.0 (94.8)
-All levels	212.2 (58.7)	189.7 (52.7)	192.7 (53.8)
Intraoperative blood loss, mean mL (SD)			
-1	700.0 (−)	349.2 (221.8)	376.2 (233.6)
-2	440.0 (260.8)	461.7 (303.4)	459.5 (296.8)
-3	650.0 (70.7)	713.2 (435.2)	707.9 (416.5)
-4	900.0 (458.3)	650.0 (353.6)	800.0 (393.7)
-5	500.0 (282.8)	–	500.0 (282.8)
-All levels	607.7 (317.4)	519.4 (355.5)	531.9 (350.1)

*Abbreviation*: *SD* Standard deviation

^a^ Among IPLF + TLIF surgeries, blood loss data were reported for 12 patients (92%) with single-level procedures, 22 patients (92%) with 3-level procedures, and 2 patients (66%) with 4-level procedures

## Discussion

This retrospective study assessed clinical outcomes using V-CBA in IPLF surgeries with and without TLIF in patients at risk for delayed union or nonunion. Recent evidence suggests that use of V-CBA leads to successful fusion, even in patients with comorbidities and lifestyle risk factors known to negatively affect fusion [14, 23]. Successful fusion (Lenke A or B ratings) was reported in 88 of 96 patients (91.7%) overall. These results concur with previous reports of successful fusion rates (98.7%) in IPLF procedures using V-CBA [10]. By comparison, successful lumbar fusion rates with the historically-preferred ICBG have been reported in a range from 54 to 90% [24–27]. Other common graft substitutes for ICBG include local autologous laminectomy bone (reported fusion rates from 65 to 93% [25, 27, 28]), and Grafton™ demineralized bone matrix (DBM) gel (Medtronic, Memphis TN) with a reported fusion rate of 52% [24].

Another commonly used substitute for ICBG is human bone morphogenetic protein-2 (rhBMP-2; Infuse™; Medtronic, Memphis TN). A retrospective study that compared use of rhBMP-2 to map 3™ CBA (M-CBA; RTI, Alachua FL) in anterior lumbar interbody fusion (ALIF) at 1 to 3 consecutive levels found that use of either product resulted in an overall fusion rate of 91% [29]. The study included patients at high risk for nonunion, including current smokers (28% for rhBMP-2 and 10% for M-CBA) and former smokers (33% for rhBMP-2 and 35% for M-CBA), although the fusion rates for these particular patients were not specified. Additionally, Overley and colleagues reported retrospective results for 78 patients undergoing minimally-invasive TLIF at an average of 1.2 levels using rhBMP-2 (39 patients) versus an MSC-based CBA (T-CBA; Trinity Evolution®; MTF Biologics, Edison NJ; 39 patients) [30]. Fusion rates assessed at 1 year were 78 and 68% for rhBMP-2 and T-CBA, respectively, in all patients, and 78 and 59%, respectively, in patients receiving single-level treatments only. Although the study included patients with diabetes (rhBMP-2 *n* = 8, T-CBA *n* = 5) and smokers (rhBMP-2 *n* = 4, T-CBA *n* = 3), specific fusion rates for these patients were not reported, and the authors found only presurgical hypertension to be a predictor of non-fusion, likely due to a high incidence of this comorbidity in the study (rhBMP-2 *n* = 17, T-CBA *n* = 21). rhBMP-2 is commonly used owing to several clinical studies that have demonstrated its efficacy in lumbar fusion surgeries compared to ICBG [31]. However, rhBMP-2 remains relatively expensive [32, 33] and has been associated with serious complications, such as wound seroma, radiculopathy, and heterotopic ossification [34, 35].

In the current study, the rate of successful fusion remained relatively consistent among patients with baseline comorbidities and lifestyle risk factors known to negatively impact fusion, with successful fusion reported in all IPLF-only patients. Among IPLF+TLIF patients in this study, successful fusion was reported in 89.8% of patients with a BMI at or above 30 kg/m^2^ (*n* = 59), which is in line with other reports of fusion rates in this population [36]. Additionally, 20 out of 22 patients (90.9%) in the IPLF+TLIF group with comorbid diabetes were successfully fused. Although the rate of fusion was slightly lower in patients currently using tobacco in this group (16 of 19 patients; 84.3%), tobacco use is known to be among the strongest predictors of non-fusion [6], and even IPLF +TLIF patients with a history of tobacco use (*n* = 53) successfully fused at a rate of 90.6% with V-CBA. All 6 patients overall with previous pseudarthrosis successfully fused even though the rates of successful fusion are also expected to be lower in these cases [37]. Further, although the majority had a BMI of greater 30 kg/m^2^, a review of the characteristics of individual patients who did not fuse (Table 2) revealed no discernable trends in comorbid diabetes, current or historical tobacco use, cancer history, or prior lumbar surgery, further supporting that V-CBA-driven fusion in lumbar surgeries does not appear to be strongly influenced by specific patient comorbidities. Finally, all patients with a Lenke C or D fusion status for whom ODI were reported indicated a reduction from preoperative ODI of between 3 and 35% in spite of their fusion status, and overall mean pre- versus postoperative ODI and VAS for applicable patients were significantly decreased.

Another relevant factor in this study was OR time, with an overall mean of 193 min at an average of 2.3 levels treated. A recent report by Kelly and colleagues of patients undergoing IPLF procedures with TLIF using rhBMP-2 at an average of 1.8 levels found a mean OR time of 235 min [38]. Further, a report by Glassman and colleagues in patients undergoing IPLF with rhBMP-2 found a mean OR time of 248 min with an average of 1.98 levels treated [26]. Thus, the present results represent an average of 42- to 55-min less OR time over these reports, in spite of a higher number of average levels fused in this study. These results were similar to those previously reported by Hall and colleagues for lumbar fusion with V-CBA (211 min), but it is important to note the difference in average number of levels treated (2.3 levels in the present study versus 4.1 levels in the Hall study) [10]. Reduction in OR time is a relevant statistic, as it could translate to substantially lower treatment costs and is known to improve clinical outcomes [39]. Additionally, overall mean intraoperative blood loss in the present study was 531.9 mL, which is within the ranges reported elsewhere for lumbar fusion surgeries [26, 38].

Although this study contributes to an understanding of successful fusion rates associated with V-CBA, it has inherent limitations. The present data represent a one-arm no-control case series and are the work of only one surgeon at a single center. However, as a retrospective study, fusion assessments were made prior to study planning and are therefore less subject to bias. Although an independent musculoskeletal radiologist assessed fusion in this study, the reliability achieved by two or more observers may have been stronger. Despite these limitations, the results of this study add important insight into the efficacy of V-CBA in spinal fusion.

## Conclusions

The results of this study suggest that V-CBA yields consistently successful fusion and significant decreases in patient-reported ODI and VAS, despite patient comorbidities and lifestyle risk factors that are known to negatively affect such bony healing.

## Data Availability

The datasets used and/or analyzed in the present study are not publicly available due to their clinical nature but are available from the corresponding author (HE) upon reasonable request and with permission from University of Toledo Medical Center.

## Abbreviations

BMI: Body mass index
CBA: Cellular bone allografts
CT: Computerized tomography
DBM: Demineralized bone matrix
ICBG: Iliac crest bone graft
IPLF: Instrumented posterior lumbar fusion
M-CBA: Map 3™ cellular bone allograft
MSCs: Mesenchymal stem cells
ODI: Oswestry Disability Index
OR: Operating room
rhBMP-2: Recombinant human bone morphogenetic protein-2
SD: Standard deviation
SEM: Standard error of the mean
T-CBA: Trinity® cellular bone allograft
TLIF: Transforaminal interbody fusion
V-CBA: ViviGen cellular bone allograft
VAS: Visual analog scale
